# 3D Joint Speaker Position and Orientation Tracking with Particle Filters

**DOI:** 10.3390/s140202259

**Published:** 2014-01-29

**Authors:** Carlos Segura, Javier Hernando

**Affiliations:** 1 Herta Security, Barcelona 08037, Spain; 2 Department of Signal Theory and Communications, Universitat Politècnica de Catalunya, Barcelona 08034, Spain; E-Mail: javier.hernando@upc.edu

**Keywords:** head pose, speaker orientation, acoustic source localization, 43.60.Jn, 43.60.Fg, 43.70.Bk, 43.60.Bf

## Abstract

This paper addresses the problem of three-dimensional speaker orientation estimation in a smart-room environment equipped with microphone arrays. A Bayesian approach is proposed to jointly track the location and orientation of an active speaker. The main motivation is that the knowledge of the speaker orientation may yield an increased localization performance and *vice versa*. Assuming that the sound produced by the speaker is originated from his mouth, the center of the head is deduced based on the estimated head orientation. Moreover, the elevation angle of the head of the speaker can be partly inferred from the fast vertical movements of the computed mouth location. In order to test the performance of the proposed algorithm, a new multimodal dataset has been recorded for this purpose, where the corresponding 3D orientation angles are acquired by an inertial measurement unit (IMU) provided by accelerometers, magnetometers and gyroscopes in the three-axes. The proposed joint algorithm outperforms a two-step approach in terms of localization and orientation angle precision assessing the superiority of the joint approach.

## 1. Introduction

In recent years, significant research efforts have been focused on developing human-computer interfaces in intelligent environments that aim to support human tasks and activities. The knowledge of the position and the orientation of the speakers present in a room constitutes valuable information allowing for better understanding of user activities and human interactions in those environments, such as the analysis of group dynamics or behaviors, deciding which is the active speaker among all present or determining who is talking to whom. In general, it can be expected that the knowledge about the orientation of human speakers would permit the improvement of speech technologies that are commonly deployed in smart-rooms. For instance, an enhanced microphone network management strategy for microphone selection can be developed based on both speaker position and orientation cues.

Very few methods have been proposed to solve the problem of speaker localization and speaker orientation estimation from acoustic signals. They differ mainly in how they approach the problem and can be coarsely classified in to two groups. The first group assumes the task of localization and orientation estimation as two separate and independent problems, working as a two-step algorithm: first locate the speaker, and then, the head orientation is estimated [1–6]. The main advantage of this approach is the simplicity and processing speed. However, the main drawback of this method is that the head orientation estimation process is highly dependent on the speaker tracking accuracy. This kind of approach does not take advantage of the fact that speaker orientation information could be used to improve the speaker localization precision.

The second group of approaches [7,8] considers the localization and the estimation of the orientation of the speaker as a joint process, which aims at improving the performance of the localization by proper weighting of the cross-correlation between microphone pairs, depending on their relative angle with the speaker, thus minimizing the degrading effects of the head orientation in the localization algorithm [9].

No previous work has been found that tackles the task of three-dimensional (3D) speaker orientation estimation with microphone arrays. This can be attributed to the fact that most smart environments have the microphones placed in nearly the same plane in order to maximize the localization performance in the *xy* coordinates, making it very difficult to estimate the head elevation angle, due to the low microphone placement diversity in the *z*-axis. Another possible cause may be the lack of acoustic databases with annotated speaker orientation and not even 3D orientation labels.

In this paper, a Bayesian approach is proposed to jointly track the location and orientation of a speaker. The main motivation is that the knowledge of the speaker orientation may yield to an increased localization performance and *vice versa*. The position and orientation of the speaker are estimated in the 3D space by means of a joint particle filter (PF) with coupled dynamic and observation models. Furthermore, the part from the vertical angle of the speaker's head can be inferred by the algorithm solely from the acoustic cues. In order to test the performance of the proposed algorithm, a new multimodal dataset has been purposely recorded, where the corresponding 3D orientation angles are acquired by an inertial measurement unit (IMU) provided by accelerometers, magnetometers and gyroscopes in the three axes. The position of the center of the head of the speaker is automatically provided by a video particle filter tracker from multiple cameras. The effectiveness of the proposed technique is assessed by means of a new proposed set of metrics derived from the multiple person tracking task [10] in Section 6.2 over the cited database, showing an increased performance for the joint PF approach in relation to the two two-step algorithms that first estimate the position and then the orientation of the speaker.

The remainder of this paper is organized as follows. In Section 2, the head rotation representation is described. Section 3 introduces the speaker localization and orientation estimation algorithms as a two-step approach. Section 4 presents an alternative two-step algorithm employing a PF at each step. Section 5 describes the joint PF. Sections 6 and 7 show the experiments and results. Finally, Section 8 gives the conclusions.

## 2. Head Rotation Representation

The parametrization of the head rotation in this work is based on the decomposition into Euler angles (*ϕ*, *θ*, *ψ*) with the *x*−*y*−*z* convention of the rotation matrix of the head into the room's frame of reference, where (*ϕ*, *θ*, *ψ*) denote the three basic rotations, one for every axis. By the *x* − *y* − *z* convention, the following rotations are chosen:

- Rotate by angle *ψ* about the head *z*-axis
- Rotate by angle *θ* about the head *y*-axis
- Rotate by angle *ϕ* about the head *x*-axis

**Figure 1.**
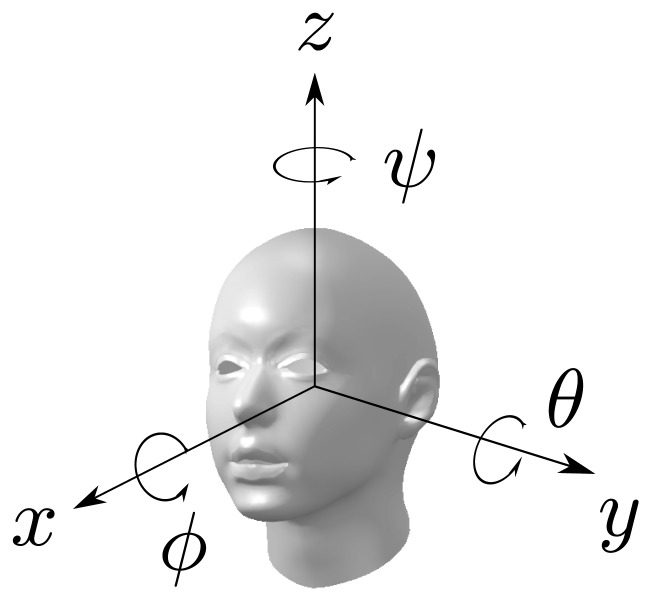
Euler angles, basic head rotations.

These rotations are shown in Figure 1.

The rotation matrix, **R**(*ϕ*, *θ*, *ψ*), is given by:

(1) $$\text{R}\left(\phi ,\theta ,\psi \right)={\text{R}}_{z}\left(\psi \right){\text{R}}_{y}\left(\theta \right){\text{R}}_{x}\left(\phi \right),$$

where:

(2) $${\text{R}}_{x}\left(\phi \right)=\left[\begin{array}{ccc} 1 & 0 & 0\\ 0 & \cos\left(\phi \right) & -\sin\left(\phi \right)\\ 0 & \sin\left(\phi \right) & \cos\left(\phi \right) \end{array}\right]$$

(3) $${\text{R}}_{y}\left(\theta \right)=\left[\begin{array}{ccc} \cos\left(\theta \right) & 0 & \sin\left(\theta \right)\\ 0 & 1 & 0\\ -\sin\left(\theta \right) & 0 & \cos\left(\theta \right) \end{array}\right]$$

(4) $${\text{R}}_{z}\left(\psi \right)=\left[\begin{array}{ccc} \cos\left(\psi \right) & -\sin\left(\psi \right) & 0\\ \sin\left(\psi \right) & \cos\left(\psi \right) & 0\\ 0 & 0 & 1 \end{array}\right]$$

The Euler angles (*ϕ*, *θ*, *ψ*) are also known as the *roll, tilt* and *pan*; or *roll, pitch* and *yaw* angles of the head. In this work, it seems not feasible to estimate the roll of the head with acoustic signals. Therefore, only the pan and tilt will be considered. Thus, the rotation of the head will be parametrized as **R**(*θ*, *ψ*) = **R**(0, *θ*, *ψ*) = **R***_z_*(*ψ*)**R***_y_*(*θ*). Nevertheless, the knowledge of the horizontal and vertical head angles, in addition to the head location, gives a good representation of the speaker in the 3D space. In order to estimate what the speaker may be referring to, the direction vector of his head in the 3D space can be computed from the rotation matrix as follows:

(5) $$\text{d}\left(\phi ,\theta \right)=\text{R}\left(\theta ,\psi \right)\;\left[\begin{array}{c} 1\\ 0\\ 0 \end{array}\right]=\left[\begin{array}{c} \text{cos}\left(\theta \right)\text{cos}\left(\psi \right)\\ \text{cos}\left(\theta \right)\text{sin}\left(\psi \right)\\ -\text{sin}\left(\theta \right) \end{array}\right]$$

## 3. Two-Step Speaker Localization-Orientation Algorithm

The two-step algorithm to estimate the location and the orientation of speakers is based on the work presented in [11]. First, the position of the speaker is estimated by the steered response power-phase transform (SRP-PHAT) algorithm and the time difference of arrival (or time delay of arrival) (TDOA) for each microphone pair with respect to the detected position is computed. In the second step, the energy of the cross-correlation nearby the estimated time delay is used as the fundamental characteristic from where to derive the speaker orientation.

### 3.1. Acoustic Source Localization

#### 3.1.1. GCC-PHAT Algorithm

In a multi-microphone environment, one of the observable clues with positional information more commonly used in acoustic localization algorithms is the time difference of arrival of the signal between microphone pairs. Consider a smart-room provided with a set of *M* microphones from which we choose *N* microphone pairs. Let x denote a ℝ^3^ position in space. Then, the time difference of arrival, *τ*_**p**,*i,j*_, of an hypothetical acoustic source located at **p** between two microphones, *i, j*, with positions **m**_i_ and **m***_j_* is:

(6) $$\tau_{\text{p},i,j}=\frac{\left\|\text{p}-{\text{m}}_{i}\|-\|\text{p}-{\text{m}}_{j}\right\|}{c},$$

where *c* is the speed of sound in air.

The cross-correlation function is well-known as a measure of the similarity between signals for any given time displacement, and ideally, it should exhibit a prominent peak in correspondence to the delay between the pair of signals [12]. A commonly used weighting function in acoustic event localization is the phase transform (PHAT), also known in the literature as cross-power-spectrum phase technique [13], which is usually considered useful in reverberant conditions. It can be expressed in terms of the inverse Fourier transform of the estimated cross-power spectrum (*G_ij_*(*f*)) with the following equation:

(7) $$R_{ij}\left(\tau \right)=\int_{-\infty }^{\infty }U\left(f_{1},f_{2}\right)\frac{G_{ij}\left(f\right)}{\left|G_{ij}\left(f\right)\right|}e^{j2\pi f\tau }df,$$

In practice, the frequency range used to compute *R_ij_*(*τ*) can be reduced to the speech-band to increase the accuracy [14], employing the rectangular band-pass filter, *U*(*f_1_*, *f*_2_), with a unitary value for frequencies *f*_1_ ≤ |*f*| ≤ *f*_2_, and zero otherwise.

The estimation of the TDOA for each microphone pair is computed as follows:

(8) τ^i,j=argmaxτRij(τ)

#### 3.1.2. SRP-PHAT Algorithm

The contributions of each microphone pair can be combined to derive a single estimation of the source position. However, in the general case, the availability of multiple TDOA estimations leads to a minimization of an over-determined and non-linear error function. A very efficient approach is the SRP-PHAT or global coherence field introduced in [14]. The SRP-PHAT algorithm performs very robustly in reverberant environments, due to the PHAT weighting, and actually, it has turned out to be one of the most successful state-of-the-art approaches to microphone array sound localization.

The basic operation of the SRP-PHAT algorithms consists of exploring the three-dimensional (3D) space, searching for the maximum of the global contribution of the PHAT-weighted generalized cross-correlations (GCC-PHAT) from all the microphone pairs. The 3D room space is quantized into a set of positions with a typical separation of 5–10 cm. The theoretical TDOA, *τ*_**p**,*i,j*_, from each exploration position to each microphone pair are precomputed and stored.

The set of GCC-PHAT functions are combined to create a spatial likelihood function (SLF) *F*(**p**), which gives a score for each position, **p**, in space by means of the following equation:

(9) $$F\left(\text{p}\right)=\sum_{i,j\in \text{S}}R_{ij}\left(\tau_{\text{p},ij}\right)$$

The estimated acoustic source location is the position of the quantized space that maximizes the contribution of the GCC-PHAT of all microphone pairs:

(10) $$\hat{\text{p}}=\underset{\text{p}}{\text{argmax}}\;F\left(\text{p}\right),$$

where 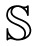 is the set of microphone pairs. Then, the TDOA for each microphone pair, τ_**p̂**,*i,j*_, is estimated using the obtained location.

### 3.2. Orientational Features

#### 3.2.1. GCC-PHAT-A

The orientational cues used in this work are based on GCC-PHAT averaged peak (GCC-PHAT-A), described in [11]. It consists on computing the energy of the cross-correlation nearby the estimated time delay by the following equation:

(11) $$\rho_{ij}\equiv \sum_{k=-\Delta }^{\Delta }{\left|w\left(k\right)R_{ij}\left(k+\tau_{\hat{\text{p}},ij}\right)\right|}^{2},$$

where *τ*_**p̂**,*ij*_ is the delay in samples and *w*(*k*) is a window with length *L* = 2Δ + 1. Different window types and lengths can be used in *w*(*k*) with satisfactory performance, as addressed in [11].

Basically, the GCC-PHAT-A measure reduces to the sum of the energy of the band-filtered PHAT-weighted cross-correlation around the estimated TDOA, and essentially, it measures the proportion of the signal between frequencies *f*_1_ and *f*_2_ that contributes to the main peak in the localization. It is also important to note that this measure is commensurable across all microphone pairs independent of microphone gains, due to the PHAT weighting and, therefore, constitutes a valuable orientational feature.

#### 3.2.2. Orientation Angle Estimation

In order to estimate the orientation of a speaker based on the GCC-PHAT-based orientational measures, a simple vectorial method is employed, similar to that described in [8]. The technique first needs the position of the active person to be known beforehand or estimated by means of the SRP-PHAT or any other source localization method. Then, the vectors, **v***_ij_*, from the speaker to the center of each microphone pair are computed, adjusting their magnitude |**v***_ij_*| to the orientational measure of the microphone pair, *ρij*. The orientational measures consists in the min-max-normalization scaled GCC-PHAT-A values, which fit in the range [−*γ*, (1 − *γ*)].

(12) $${\bar{\rho }}_{ij}=\frac{\left(\rho_{ij}-\rho_{\text{min}}\right)}{\left(\rho_{ij}-\rho_{\text{max}}\right)}-\gamma$$

(13) $${\text{v}}_{ij}={\bar{\rho }}_{ij}\frac{\hat{\text{p}}-\left({\text{m}}_{i}+{\text{m}}_{j}\right)/2}{\left\|\hat{\text{p}}-\left({\text{m}}_{i}+{\text{m}}_{j}\right)/2\right\|},$$

where *ρ_min_* and *ρ_max_* are the minimum and maximum value of the set of *ρ_ij_*. Min-max normalization retains the original distribution of values, except for a scaling factor and transforms all values into the desired range [15]. The min-max normalization models the fact that the microphone pairs with the lowest orientational cue value are probably behind the speaker, and by giving those pairs a negative value, its resulting vector would help point to the correct direction. In our experiments, we obtained good results with *γ* = 0.3.

The sum of the vectors formed by all the orientational measures of each microphone pair is considered the estimated head direction, **v***_sum_*, as follows:

(14) $${\text{v}}_{\text{sum}}=\sum_{i,j\in \text{S}}{\text{v}}_{ij}$$

The estimated head orientation angle, *ψ̂*, is computed as the angle of the projection of **v***_sum_* in the *xy*-plane with the *x*-axis.

## 4. Two-Step Particle Filter Tracking

In this section, a two-step approach to estimate the location and orientation of the speaker is proposed, employing a particle filter in each stage, which is introduced here to enable a fair comparison with the joint particle filter approach.

### 4.1. Particle Filter Tracking

The concept of tracking can be defined as the recursive estimation of the hidden state of a target based on the partial observations at every time instant. Assuming that the evolution of the state sequence is defined by a Markov process of first order, the dynamics of the state can be described by the transition equation:

(15) $${\text{x}}_{k}={\text{f}}_{k}\left({\text{x}}_{k-1},{\text{v}}_{k-1}\right)$$

where **f***_k_* is a possibly non-linear function of the previous state, **x**_*k*−1_, and an independent and identically distributed (i.i.d.) process noise, **v**_*k*−1_. At every time instant, *k*, the observation of the state, **x***_k_*, is defined by the observation equation:

(16) $${\text{z}}_{k}={\text{h}}_{k}\left({\text{x}}_{k},{\text{n}}_{k}\right)$$

where, again, **h***_k_* is, in general, a non-linear function of the state and an i.i.d. measurement noise sequence, **n***_k_*.

Tracking aims to estimate **x***_k_* based on the set of all available measurements **z**_1:_*_k_* = {**z**_i_, *i* = 1,…, *k*} up to time *k*. One solution is to use the Bayesian approach to reconstruct the probability density function (pdf) of **x***_k_* given all the data, **z**_1:*k*_, up to time *k*, or in a compact notation, *p*(**x***_k_*∣**z**_1:_*_k_*). The pdf, p(**x***_k_*∣**z**_1:_*_k_*), is known as the *posterior density* and contains all statistical information gathered by the measurements up to time *k*. The posterior density may be obtained recursively by means of the Bayesian approach based on two fundamental iteration steps, namely, prediction and update.

In the prediction step, the prior pdf, *p*(**x***_k_*∣**z**_1:__*k*−1_), is obtained making use of the *transition* pdf, *p*(**x***_k_*∣**x**_*k*−1_), which is derived from transition Equation (15):

(17) $$p\left({\text{x}}_{k}|{\text{z}}_{1:k-1}\right)=\int p\left({\text{x}}_{k}|{\text{x}}_{k-1}\right)p\left({\text{x}}_{k-1}|{\text{z}}_{1:k-1}\right)d{\text{x}}_{k-1}$$

In the update stage, the new measurement, **z***_k_*, is used to update the prior pdf via the Bayes' rule and obtain the required posterior density of the current state:

(18) $$p\left({\text{x}}_{k}|{\text{z}}_{1:k}\right)=\frac{p\left({\text{z}}_{k}|{\text{x}}_{k}\right)p\left({\text{x}}_{k}|{\text{z}}_{1:k-1}\right)}{p\left({\text{z}}_{k}|{\text{z}}_{1:k-1}\right)}$$

where the denominator:

(19) p(zk∣z1:k−1)=∫p(zk∣xk)p(xk∣z1:k−1)dxk

is a normalizing constant, which depends on the pdf, *p*(**z***_k_*∣**x***_k_*), defined by observation Equation (16).

Particle filters (PF) [16] approximate the Bayesian filter approach by representing the probability distribution recursively with a finite set of samples, known as particles, that are updated according to their measured likelihood for a given dynamical and observational model. Applications of PF to acoustic localization can be found in [17–19] with a comprehensive research in [20].

Let ${\left\{{\text{x}}_{k}^{i}\right\}}_{i=1}^{N_{s}}$ denote a set of *N_s_* random samples of the state with associate weights ${\left\{w_{k}^{i}\right\}}_{i=1}^{N_{s}}$ normalized such that $\sum_{i}w_{k}^{i}=1$. Then, the posterior density, *p*(**x***_k_*∣**z**_1:_*_k_*), can be approximated as:

(20) $$p\left({\text{x}}_{k}|{\text{z}}_{1:k}\right)\approx \sum_{i=1}^{N_{s}}w_{k}^{i}\delta \left({\text{x}}_{k}-{\text{x}}_{k}^{i}\right)$$

Considering that the samples, ${\text{x}}_{k}^{i}$ are drawn from a sampling distribution, $q\left({\text{x}}_{k}|{\text{x}}_{k-1}^{i},{\text{z}}_{k}\right)$ called *importance density*, and taking some widely accepted assumptions [16], the weights can be computed recursively by the following expression:

(21) $$w_{k}^{i}\propto w_{k-1}^{i}\frac{p\left({\text{z}}_{k}|{\text{x}}_{k}^{i}\right)p\left({\text{x}}_{k}|{\text{x}}_{k-1}^{i}\right)}{q\left({\text{x}}_{k}|{\text{x}}_{k-1}^{i},{\text{z}}_{k}\right)}$$

In the literature regarding other domains, some techniques aim at constructing efficient importance density functions through Markov Chain Monte Carlo methods [21] or exploiting independence among variables in the state space using Rao-Blackwellized particle filters [22]. Although there is a large number of methods to compute the associated particle weights, one approach that is the most largely accepted, in part for its convenience, is to choose the importance density to be the prior:

(22) $$q\left({\text{x}}_{k}|{\text{x}}_{k-1}^{i},{\text{z}}_{k}\right)=p\left({\text{x}}_{k}|{\text{x}}_{k-1}^{i}\right)$$

reducing the weight recursion to:

(23) $$w_{k}^{i}\propto w_{k-1}^{i}p\left({\text{z}}_{k}|{\text{x}}_{k}^{i}\right)$$

A common problem with the PF is the degeneracy phenomenon, where, after a few iterations, all the weight concentrates in just one particle, and the rest of the particles have almost zero contribution to the approximation of the posterior. A measure of the degeneracy of the PF is the *effective sample size* introduced in [23] and [24], defined as:

(24) $$\hat{N_{\text{eff}}}=\frac{1}{\sum_{i}^{N_{s}}{\left(w_{k}^{i}\right)}^{2}}$$

where $\hat{N_{\text{eff}}}\leq N_{s}$ and a small $\hat{N_{\text{eff}}}$ is a symptom of severe degeneracy. Although this problem could be tackled by using a very large *N_s_*, a common approach, whenever a significant degeneracy is observed, is to make use of particle resampling techniques, which consist of discarding the particles with lower weight and proportionally replicating those with a higher one, while still representing the posterior density.

The best estimation of the state at time *k*, **x̂***_k_*, is derived based on the discrete approximation of Equation (20). The most common solution is the Monte Carlo approximation of the expectation:

(25) $${\hat{\text{x}}}_{k}=\text{E}\left[{\text{x}}_{k}|{\text{z}}_{1:k}\right]\approx \sum_{i=1}^{N_{s}}w_{k}^{i}{\text{x}}_{k}^{i}$$

The design parameters of the PF are the state model, the dynamical model and the observational model, which are defined in the following sections.

### 4.2. Location Tracking

#### 4.2.1. State and Dynamical Models

A common approach is to characterize the human movement dynamics as a *Langevin process* [25], since it is reasonably simple and has been proven to work well in practical applications [19,25]. In this case, the state variable, **x***_k_*, is defined as:

(26) $${\text{x}}_{k}=\left[\begin{array}{c} {\text{p}}_{k}\\ {\dot{\text{p}}}_{k} \end{array}\right]$$

where **p***_k_* = [*x_k_ y_k_ z_k_*]*^T^* denotes the position and **ṗ***_k_* = [*ẋ_k_ ẏ_k_ ż_k_*]*^T^* denotes the velocity of the target. The addition of the velocity component in the state variable aims to improve the representation of the movement dynamics.

For the sake of simplicity, consider the Langevin process in the *x*-coordinate as follows:

(27) $$x_{k}=x_{k-1}+T{\dot{x}}_{k}$$

(28) $${\dot{x}}_{k}=a{\dot{x}}_{k-1}+\sigma_{x}n_{x}$$

where *n_x_* ∼ 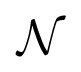
(0, 1) is a normally distributed random variable, *T* is the time step unit between consecutive updates of the state vector and the two constants are defined as:

(29) $$a=e^{-\beta T}$$

(30) $$\sigma_{x}=\upsilon_{x}\sqrt{1-a^{2}}$$

where *υ* denotes the steady-state root mean square velocity and *β* is the rate constant. The motion model in the *x* and *y* coordinates is assumed to be independent and identically distributed, which yields to identical model parameters in both coordinates. The random variable, *n_z_*, for the *z*-axis is set to have a normal distribution with a very low variance, $\sigma_{z}^{2}$. Equations (27) and (28) can be rewritten following the form of transition Equation (15):

(31) $${\text{x}}_{k}=\underset{\text{F}}{\underset{︸}{\left[\begin{array}{cccccc} 1 & 0 & 0 & aT & 0 & 0\\ 0 & 1 & 0 & 0 & aT & 0\\ 0 & 0 & 1 & 0 & 0 & aT\\ 0 & 0 & 0 & a & 0 & 0\\ 0 & 0 & 0 & 0 & a & 0\\ 0 & 0 & 0 & 0 & 0 & a \end{array}\right]}}\;{\text{x}}_{k-1}+{\text{v}}_{k-1}$$

with **v**_*k*−1_ characterized as a zero-mean Gaussian noise variable with covariance matrix **Q**_*k*−1_:

(32) $${\text{v}}_{k-1}∼\text{N}\;\left[\begin{array}{c} 0\\ 0\\ 0\\ 0\\ 0\\ 0 \end{array}\right],\underset{{\text{Q}}_{k-1}}{\underset{︸}{\left[\begin{array}{cccccc} \sigma_{x}^{2}T^{2} & 0 & 0 & 0 & 0 & 0\\ 0 & \sigma_{y}^{2}T^{2} & 0 & 0 & 0 & 0\\ 0 & 0 & \sigma_{z}^{2}T^{2} & 0 & 0 & 0\\ 0 & 0 & 0 & \sigma_{x}^{2} & 0 & 0\\ 0 & 0 & 0 & 0 & \sigma_{y}^{2} & 0\\ 0 & 0 & 0 & 0 & 0 & \sigma_{z}^{2} \end{array}\right]}}$$

#### 4.2.2. Observational Model

The particle filter approach requires the definition of the likelihood function, $p\left({\text{z}}_{k}|{\text{x}}_{k}^{i}\right)$ in order to update the weight of every particle. In this case, the observation, **z***_k_*, is not limited to the estimated source location [19], and the full SRP-PHAT SLF generated by Equation (9) or a modification thereof can be employed [26]. Other works [17] construct the likelihood function employing solely the TDOA estimations.

In this work, the localization likelihood is derived from a spatial likelihood function *F*(**p**) obtained by the SRP-PHAT algorithm with the PHAT-weighted cross-correlation smoothed by the convolution with a triangular window, Ω(*τ*), of five samples:

(33) $${\tilde{R}}_{ij}\left(\tau \right)={\left(R_{ij}^{2}\left(\tau \right)*\Omega \left(\tau \right)\right)}^{1/2}$$

(34) $$F\left(\text{p}\right)={\left(\sum_{i,j\in \text{S}}{\tilde{R}}_{ij}\left(\tau_{\text{p},i,j}\right)\right)}^{2}$$

Given the iterative nature of the PF, this smoothed SLF enables a faster convergence of the particles to its global maximum, while avoiding being trapped around local maxima. Since the position that maximizes *F*(**p**) determines the most probable location of the sound source, the localization observation likelihood function is constructed from the estimated position of the speaker's mouth, **t***_k_*, and the SLF:

(35) $$p\left({\text{z}}_{k,\text{loc}}|{\text{x}}_{k}\right)=F\left({\text{t}}_{k}\right),$$

where **z***_k,loc_* denotes the observation of the localization.

The likelihood function, *F*(**p**), is usually precomputed for a discrete set of space positions for every audio frame in order to gain speed in the evaluation of *p*(**z***_k,loc_*∣**x***_k_*) in the case of a PF with a large number of particles, at the expense of localization precision. In this work, the quantization step is set to 5 cm.

### 4.3. Orientation Tracking

#### 4.3.1. State and Dynamical Models

The state vector of the particle filter used to estimate the orientation consists only of the pan angle and the dynamical model as follows:

(36) $${\text{x}}_{k}=\psi_{k}$$

(37) $$\psi_{k}=\psi_{k-1}+n_{\psi }$$

where $n_{\psi }∼\text{N}\left(0,\sigma_{\psi }^{2}\right)$ is a normally distributed random variable.

The state head direction vector in 3D space **d***_k_*(*ψ_k_*) = [*cos*(*ψ_k_*) *sin*(*ψ_k_*) 0]^*T*^.

#### 4.3.2. Observational Model

The orientation likelihood is obtained from the GCC-PHAT averaged peak features described in Section 3.2. A vector, **v**_*n*_, is created from the estimated speaker's position, **p***_k_*, to the center of each microphone pair, adjusting their magnitude |**v**_*n*_| to the normalized orientational measure of the microphone pair as defined in Section 3.2.2. The orientation observation is formed by the resulting vector, **v***_sum_*, of the vectorial sum of **v***_n_*. The orientation likelihood function is then defined as the scalar product of the state head direction vector and the normalized resulting vector as follows:

(38) $$p\left({\text{z}}_{k,\text{ori}}|{\text{x}}_{k}\right)={\left(\frac{\langle {\text{d}}_{k}\left(\psi_{k}\right),\frac{{\text{v}}_{\text{sum}}^{T}}{\left|{\text{v}}_{\text{sum}}\right|}\rangle +1}{2}\right)}^{C\left|{\text{v}}_{\text{sum}}\right|}$$

where 〈·,·〉 denotes the inner product, **z***_k,ori_* is the observation of the orientation and *C* is a constant to control the width of the observation probability function.

The scalar product of the two unitary vectors is scaled into the range [0, 1] to better resemble a likelihood function. The exponent, *n*|**v***_sum_*|, is used as a *confidence factor* for the orientational observation, with the constant, *n*, set empirically to four. The magnitude of the observation vector, |**v***_sum_*|, models the likelihood function, where a very small value of the vector length yields to the constant likelihood function independent of the state. On the other hand, higher values of the observation vector magnitude will narrow the likelihood function to observation angles close to the state angle.

## 5. Joint Localization-Orientation Particle Filter Tracker

In this work, a particle filter approach is proposed to jointly track the location and orientation of a speaker. The main motivation is that the knowledge of the speaker orientation may yield to an increased localization performance and *vice versa*. The position and orientation of the speaker are estimated in the 3D space by means of a joint particle filter with coupled dynamic and observation models. The proposed system makes the assumption that the voice of a speaker is produced around the mouth, and the knowledge about the orientation yields to a better estimate of the head position. On the other hand, in this work, it is proposed to assume that the person movement is dependent on his orientation and *vice versa*. Next sections describe the proposed state and coupled dynamic and observation models.

### 5.1. State Model

The state of the particles is composed by the position of the center of the speaker's head **p***_k_* = [*x_k_ y_k_ z_k_*]*^T^*, the velocity of the speaker **ṗ***_k_* = [*ẋ_k_ ẏ_k_ ż_k_*]*^T^* and the tilt and pan of his head.

(39) $${\text{x}}_{k}=\left[\begin{array}{c} {\text{p}}_{k}\\ {\dot{\text{p}}}_{k}\\ \psi_{k}\\ \theta_{k} \end{array}\right]$$

The estimated head rotation at any time is defined by:

(40) $${\text{R}}_{k}\left(\theta_{k},\psi_{k}\right)=\left[\begin{array}{ccc} \cos\left(\theta_{k}\right)\cos\left(\psi_{k}\right) & -\sin\left(\psi_{k}\right) & \sin\left(\theta_{k}\right)\cos\left(\psi_{k}\right)\\ \cos\left(\theta_{k}\right)\sin\left(\psi_{k}\right) & \cos\left(\psi_{k}\right) & \sin\left(\theta_{k}\right)\sin\left(\psi_{k}\right)\\ -\sin\left(\theta_{k}\right) & 0 & \cos\left(\theta_{k}\right) \end{array}\right]$$

The estimation of the position of the speaker's mouth **t***_k_* is determined at every instant by the state vector, and it is synthesized from the head center position and the rotation angles as follows:

(41) $${\text{t}}_{k}={\text{p}}_{k}+{\text{R}}_{k}\left(\theta_{k}+\alpha ,\psi_{k}\right)\;{\left[\begin{array}{ccc} r & 0 & 0 \end{array}\right]}^{T}$$

where it has been assumed that the mouth lies at *r* distance from the head center with an inclination angle of *α*. A preliminary radius of *r* = 15 cm and an inclination of *α* = 45 degrees have been set experimentally.

The state head direction vector in the 3D space, **d***_k_*(*θ_k_, ψ_k_*), is computed, rotating the head direction vector in the head coordinate reference to the 3D space reference:

(42) $${\text{d}}_{k}\left(\theta_{k},\psi_{k}\right)={\text{R}}_{k}^{i}\left(\theta_{k},\psi_{k}\right)\;{\left[\begin{array}{ccc} 1 & 0 & 0 \end{array}\right]}^{T}$$

### 5.2. Dynamical Model

Similarly to Section 4.2.1, a *Langevin process* is chosen to characterize the speaker movement dynamics. Usually, the motion model in the *x* and *y* coordinates is assumed to be independent and identically distributed, which yields to identical model parameters in both coordinates. However, in this work, it is assumed that the movement in the *x* and *y* coordinates is dependent on the *pan* orientation angle of the person. It is expected as a more probable event that the speaker moves to his forward direction than to his sideways or backward directions. This is modeled as a Rayleigh distribution probability in the speaker's forward direction and a normal distribution in his sideways direction. The Rayleigh distribution, 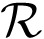
(0,1), is scaled and centered in order to have a zero mean expectation and unity variance. The variance of the distributions determined by the *σ* factor is also different for the forward and sideways directions.

(43) $$n_{\text{forward}}∼ℛ\left(0,\sigma_{\text{forward}}^{2}\right)$$

(44) $$n_{\text{sideway}}∼N\left(0,\sigma_{\text{sideway}}^{2}\right)$$

The random variable, *n_x_*, from Equation (28) for the *x* and *y* coordinates are obtained by the rotation of *n_forward_* and *n_sideway_* by the *pan* angle *ψ_k_*:

(45) $$\left[\begin{array}{c} n_{x}\\ n_{y} \end{array}\right]=\left[\begin{array}{cc} \cos\left(\psi_{k}\right) & -\sin\left(\psi_{k}\right)\\ \sin\left(\psi_{k}\right) & \cos\left(\psi_{k}\right) \end{array}\right]\;\left[\begin{array}{c} n_{\text{forward}}\\ n_{\text{sidway}} \end{array}\right]$$

The random variable, *n_z_*, for the *z*-axis is set to have a normal distribution with a very low variance, $\sigma_{z}^{2}$.

In this work, the horizontal orientation angle of the speaker is assumed to be dependent on his velocity. It is expected that the faster the person moves, the more probable it is that the person is looking to his moving direction. This is modeled by predicting the next state head direction as the weighted sum of the current state head direction vector in the *xy* plane and the normalized moving direction vector plus a normally distributed random variable, **n***_d_*, where the weight factor, *α_ψ_*, depends on the person's velocity and the maximum expected velocity, *υ_max_*, as follows:

(46) $$\alpha_{\psi }=\frac{\left|{\dot{\text{p}}}_{k-1}\right|}{\upsilon_{\text{max}}}$$

(47) $$\left[\begin{array}{c} d_{x}\\ d_{y}\\ d_{z} \end{array}\right]=\left(1-\alpha_{\psi }\right){\text{d}}_{k-1}\left(0,\psi_{k-1}\right)+\alpha_{\psi }\frac{{\dot{\text{p}}}_{k-1}}{\left|{\dot{\text{p}}}_{k-1}\right|}+{\text{n}}_{d}$$

(48) $${\text{n}}_{d}∼\text{N}\;\left(\left[\begin{array}{c} 0\\ 0\\ 0 \end{array}\right],\left[\begin{array}{ccc} \sigma_{d}^{2} & 0 & 0\\ 0 & \sigma_{d}^{2} & 0\\ 0 & 0 & 0 \end{array}\right]\right)$$

*d_x_*, *d_y_* and *d_z_* being the *x*, *y* and *z* components of **d**_*k*−1_.

Finally, the next state *yaw* orientation is the angle formed by the *y* and *x* components of the head direction:

(49) $$\psi_{k}=\text{arctan}(d_{y},d_{x})$$

The *pitch* orientation angle recursion equation, assuming independence with other state variables, is defined as:

(50) $$\theta_{k}=\alpha_{\theta }\theta_{k-1}+n_{\theta }$$

where *α_θ_* is a forgetting factor accomplishing |*α_θ_*| ≤ 1 and $n_{\theta }∼N\left(0,\sigma_{\theta }^{2}\right)$ is a normally distributed random variable. *The pitch*, *θ_k_*, determines the height of the mouth of the speaker in relation to the head center position. The variables, *α_θ_*, *n_θ_* and *n_z_*, are adjusted, so that short-term vertical head movements are inferred by *θ_k_*, whereas long-term smooth head height changes are incorporated into the state head height, due to the forgetting factor.

### 5.3. Observational Model

The observation likelihood, *p*(**z***_k_*∣**x***_k_*), is composed from the localization, **z***_k,loc_*, from Equation (35) and orientation **z***_k,ori_* from Equation (38) feature observations as follows:

(51) $$p({\text{z}}_{k}|{\text{x}}_{k})=p({\text{z}}_{k,\text{loc}}|{\text{x}}_{k})p({\text{z}}_{k,\text{ori}}|{\text{x}}_{k})$$

where it is assumed that these observations are conditionally independent, given the current state, **x***_k_*.

## 6. Experiments

### 6.1. Experimental Setup and Database Description

The joint PF tracker performance will be compared with the two two-step algorithms introduced in Sections 3.2.2. and 4 in the task of estimating the position and orientation of the speaker's head. Since the two-step approaches are only able to estimate the horizontal orientation angle, the pitch and roll hypothesis are set to 0 for all time frames. The comparison with the two-step PF approach assesses that the performance increase obtained by the joint method is due to the joint dynamic and observation models and not the filtering itself.

**Figure 2.**
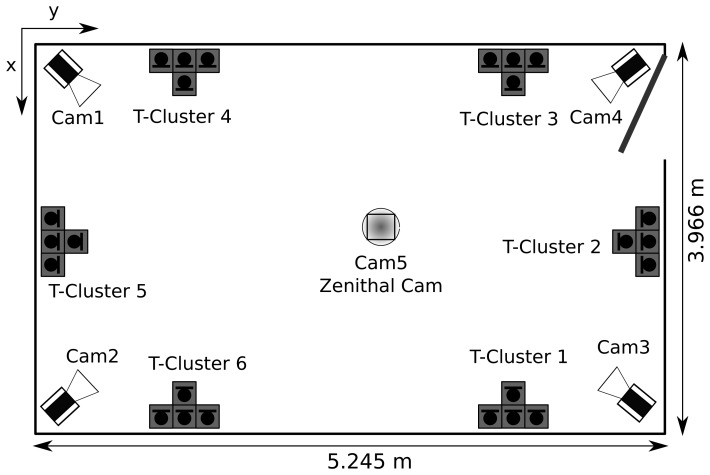
Smart-room sensor setup used in this database, with 5 cameras (Cam1-Cam5) and 6 T-shaped microphone clusters (T-Cluster 1 -6).

The performance of the proposed head orientation estimation algorithm was evaluated using a purposely recorded database collected in the smart-room at the Universitat Politècnica de Catalunya. It is a meeting room equipped with several multimodal sensors, such as microphone arrays, table-top microphones and fixed or pan-tilt-zoom video cameras. The room dimensions are 3, 966×5, 245×4,000 mm, which correspond to the *x*, *y* and *z* coordinates, respectively, and its measured reverberation time is approximately 400 ms. A schematic figure of the room setup can be observed in Figure 2.

**Figure 3.**
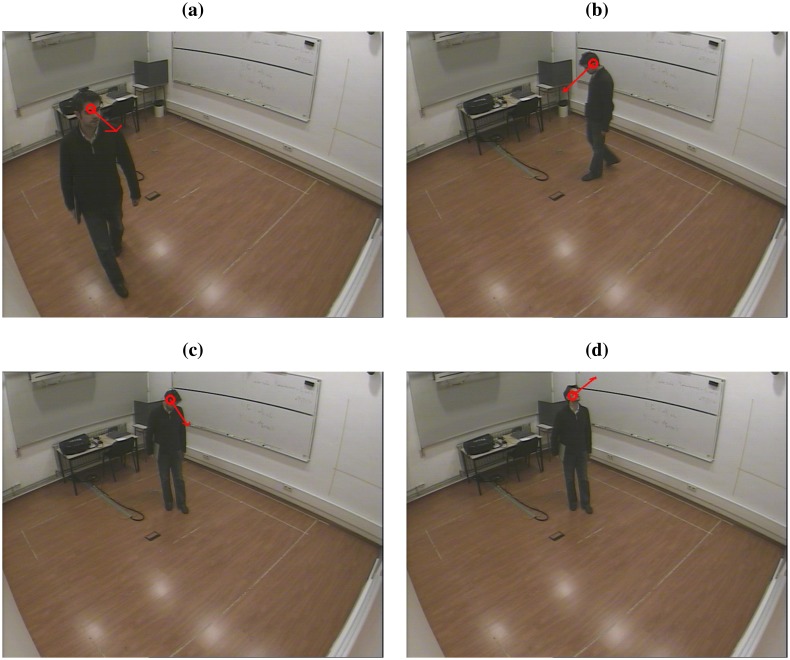
Single person dataset snapshots, with superposed head position and rotation annotations.

The database is composed of one single person dataset involving the recording of multi-microphone audio, multi-camera video and IMU data for seven people moving freely in a smart room speaking most of the time and another multi-person dataset consisting in the recording of a group discussion with four participants. Only the simple person dataset will be considered in this work, since it is oriented toward the person tracking task, while the multi-person dataset is oriented toward the group analysis task. A sample of the database is shown in Figure 3.

The ground truth provided by the database consists in the annotations of the center of the head and the Euler rotation angles of every participant. The center of the head was obtained automatically by means of a multi-camera video PF tracker and the Euler orientation angles are acquired by an inertial measurement unit (IMU) provided by accelerometers, magnetometers and gyroscopes in the three axes.

### 6.2. Metrics

The metrics proposed in [10] for acoustic and audiovisual person-tracking are considered for evaluation and comparison purposes. These metrics have been used in international evaluation contests [27] and have been adopted by several research projects, such as the European Computers in the Human Interaction Loop (CHIL) [28] or the U.S. VACE [29] thus, they allow an objective and fair comparison with other acoustic tracking methods and with methods from other modalities.

In [10], two metrics are defined for an acoustic and audiovisual person-tracking task. Multiple object tracking precision (MOTP), which shows the trackers ability to estimate precise object positions, whereas multiple object tracking accuracy (MOTA) expresses the performance for estimating the correct number of objects and keeping to consistent trajectories. Additionally, the acoustic multiple object tracking accuracy (A-MOTA) score is defined for the acoustic tracking task, evaluated only for the active speaker at each time instant, *k*. A new metric is proposed in this work, multiple head orientation tracking precision (MHOTP), which determines the performance for estimating the head orientation of multiple persons.

#### 6.2.1. Multiple Object Tracking Accuracy (MOTA) (%)

This is the accuracy of the tracker when it comes to keeping correct correspondences over time, estimating the number of people, recovering tracks, *etc.*, the tracker, false positives, misses and mismatches, over all frames, divided by the total number of ground truth points.

(52) $$\text{MOTA}=1-\frac{\sum_{k}ms_{k}+\sum_{k}fp_{k}+\sum_{k}mm_{k}}{\sum_{k}g_{k}}$$

where *ms_k_*, *fp_k_* and *mm_k_* denote, respectively, the number of misses, false positives and mismatches, and *g_k_* is the number of ground truth objects at time instant *k*. A distance threshold of 1 m is used to associate a track with the ground truth. Distances above this threshold will be treated as either false positives or mismatches. A more detailed description of this metric can be found in [10].

#### 6.2.2. Multiple Object Tracking Precision (MOTP) (mm)

This is the precision of the tracker when it comes to determining the exact position of a tracked person in the room.

(53) $$\text{MOTP}=\frac{\sum_{i,k}d_{i,k}}{\sum_{k}c_{k}}$$

where *c_k_* is the number of correspondence matches found for time frame *k* and *d_i,k_* is the distance between the ground truth position and its corresponding hypothesis.

#### 6.2.3. Multiple Head Orientation Tracking Precision (MHOTP) (degrees)

This is the precision of the tracker when it comes to determining the exact orientation of a tracked person in the room. It is the Euclidean angle error for matched *ground truth-hypothesis* pairs over all frames, averaged by the total number of matches made. It shows the ability of the tracker to estimate the correct orientation and is independent of its tracking accuracy. The Euclidean angle is computed as the angle between the estimated head direction vector, **d̂**(*θ̂, ψ̂*), and the ground truth vector, **d**(*θ, ψ*). The multiple head orientation tracking precision can be also detailed by three sub-metrics, which account for the angle error in every axis.

(54) $${\text{MHOTP}}_{\psi }=\frac{\sum_{i,k}\left|{\hat{\psi }}_{i,k}-\psi_{i,k}\right|}{\sum_{k}c_{k}}$$

(55) $${\text{MHOTP}}_{\theta }=\frac{\sum_{i,k}\left|{\hat{\theta }}_{i,k}-\theta_{i,k}\right|}{\sum_{k}c_{k}}$$

(56) $${\text{MHOTP}}_{\phi }=\frac{\sum_{i,k}\left|{\hat{\phi }}_{i,k}-\phi_{i,k}\right|}{\sum_{k}c_{k}}$$

(57) $$\text{MHOTP}=\frac{\sum_{i,k}\text{arccos}(\langle \text{d}(\theta_{i,k},\psi_{i,k}),\text{d}({\hat{\theta }}_{i,k},{\hat{\phi }}_{i,k})\rangle )}{\sum_{k}c_{k}}$$

(58) $$\text{d}(\theta ,\psi )=\left[\begin{array}{c} \cos(\theta )\cos(\psi )\\ \cos(\theta )\sin(\psi )\\ -\sin(\theta ) \end{array}\right]$$

where *ϕ_i,k_*, *θ_i,k_* and *ψ_i,k_* are the ground truth Euler angles for the target, *i*, at the time instant, *k*, and *ϕ̂_i,k_*, *θ̂_i,k_* and *ψ̂_i,k_* are the estimated Euler angles for the corresponding hypothesis.

## 7. Results

Experiments were conducted over the cited database to compare the performance of the joint PF tracker and the two two-step approaches. A tight relationship between the tracking accuracy (MOTA) and precision (MOTP and MHOTP) has been observed in the three algorithms, since it is possible to output a localization and orientation hypothesis only when the confidence of the algorithm is above a threshold and achieve a high precision at the expense of tracking accuracy, and *vice versa*. In order to ensure a fair comparison between the three algorithms, the peak value of the SLF is selected as the confidence for all methods, where a sweep threshold parameter is used to obtain the curve of all possible accuracy and precision results.

**Figure 4.**
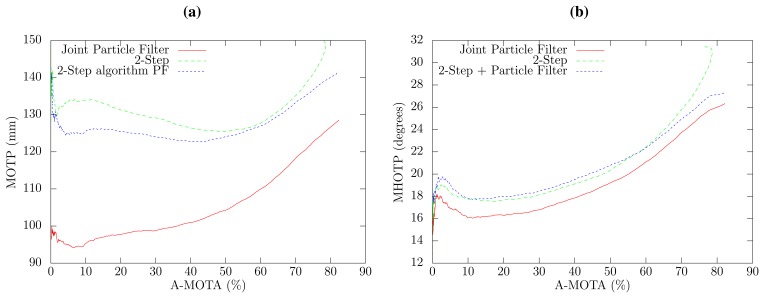
Curve of all possible tracking accuracy (acoustic multiple object tracking accuracy (A-MOTA)), localization tracking precision (multiple object tracking precision (MOTP)) (a) and 3D orientation angle precision (multiple head orientation tracking precision (MHOTP)) (b) results, employing a sweep threshold parameter on the algorithm confidence.

Figure 4a shows the position tracking error in relation to the tracking accuracy for the three methods. The two-step PF approach is slightly better than the two-step algorithm. However, the proposed joint PF approach obtains a notable performance increase in the localization precision with respect to the two-step PF approach, that ranges from 7% to 24% error reduction depending on the A-MOTA working point. This increased localization precision is due to the fact that the database position annotations correspond with the head center position (this is a general fact for almost all tracking databases), whereas the acoustic localization algorithm detects the position of the mouth of the speaker. The proposed joint algorithm takes advantage of the knowledge of the mouth position and head orientation to estimate the center of the head, thus obtaining better localization results. Two A-MOTA working points have been selected to show numerical values, which can be observed in Tables 1 and 2.

**Figure 5.**
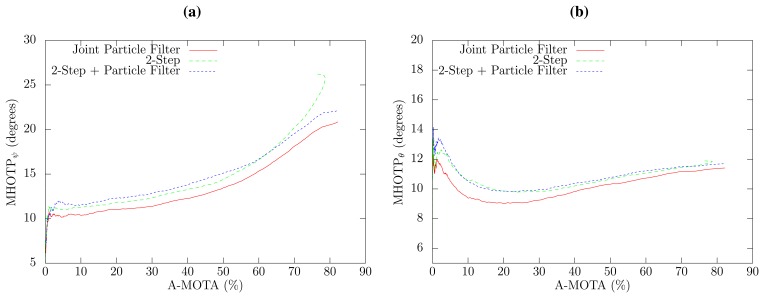
Curve of all possible tracking accuracy (A-MOTA), horizontal orientation angle precision (MHOTP*_ψ_*) (a) and vertical orientation angle precision (MHOTP*_θ_*) (b) results, employing a sweep threshold parameter on the algorithm confidence.

The overall precision of the estimation of 3D direction of the head in relation to the tracking accuracy is shown in Figure 4b for all methods. The joint PF approach exhibits an overall reduction of 1.4 degrees in the 3D angle estimation error with respect to the two-step approaches, which have a very similar performance. The 3D angle error can be split in the horizontal and vertical angle error, which are shown in Figure 5, respectively. The proposed joint method has a horizontal angle error reduction of 8.2% to 9.1%, depending on the selected confidence threshold in comparison to both two-step approaches, which, again, have a very similar angle error. Interestingly, the results obtained for the vertical angle, which are similar to the localization results, have better precision when only high confidence SLF values are employed. This can be explained by the fact that the proposed method estimates the elevation angle from the small term height changes produced by the acoustic localization algorithm and that high confidence SLF values provide a more accurate acoustic source position.

## 8. Conclusions

A PF approach for joint head position and 3D orientation estimation has been presented in this article. Experiments conducted over the purposely recorded database with Euler angles and head center annotations for seven different people in a smart room showed an increased performance for the joint PF approach in relation to two two-step algorithms that first estimate the position and then the orientation of the speaker. Both two-step approaches have a very similar angle estimation error, with a small increase in the localization precision (MOTP) for the two-step PF. The proposed joint algorithm outperforms both two-step algorithms in terms of localization precision and orientation angle precision (MHOTP), assessing the superiority of the joint approach. Furthermore, by means of the definition of a joint dynamical model, part of the the elevation angle of the head is inferred by the algorithm. Future work will be devoted to extending the joint PF to track multiple speakers and to study the fusion with video approaches with a focus on 3D orientation estimation.

## Figures and Tables

**Table 1.** Tracking performance joint and two-step approaches for an A-MOTA working point of 10%. PF, particle filter.

System	MOTP	MHOTP	MHOTP*_ψ_*	MHOTP*_θ_*
2-Step	133.94 mm	17.76°	11.27°	10.63°
2-Step PF	125.58 mm	17.84°	11.53°	10.53°
Joint PF	95.30 mm	16.06°	10.38°	9.39°

**Table 2.** Tracking performance joint and two-step approaches for an A-MOTA working point of 75%.

System	MOTP	MHOTP	MHOTP*_ψ_*	MHOTP*_θ_*
2-Step	140.86 mm	28.04°	22.64°	11.54°
2-Step PF	136.62 mm	26.25°	21.01°	11.58°
Joint PF	122.67 mm	25.08°	19.60°	11.30°
